# Impact of fiducial markers placement on the delineation of target volumes in radiation therapy for oesophageal cancer: FIDUCOR study

**DOI:** 10.3389/fonc.2022.1012712

**Published:** 2022-10-28

**Authors:** Philippe Rochigneux, Marguerite Tyran, Aurélie Autret, Leonor Lopez Almeida, Jérôme Guiramand, Marjorie Ferre, Brice Chanez, Emmanuel Mitry, Jean Philippe Ratone, Marc Giovannini, Agnès Tallet, Laurence Moureau-Zabotto, Fabrice Caillol

**Affiliations:** ^1^ Department of Medical Oncology, Paoli-Calmettes Institute, Marseille, France; ^2^ Department of Radiation Oncology, Paoli-Calmettes Institute, Marseille, France; ^3^ Department of Biostatistics, Paoli-Calmettes Institute, Marseille, France; ^4^ Department of Clinical Research, Paoli-Calmettes Institute, Marseille, France; ^5^ Department of Surgery, Paoli-Calmettes Institute, Marseille, France; ^6^ Department of Biophysic, Paoli-Calmettes Institute, Marseille, France; ^7^ Department of Gastroenterology, Paoli-Calmettes Institute, Marseille, France

**Keywords:** oesophageal cancer, radiation therapy (radiotherapy), fiducial markers (FMs), target volumes, endoscopic ultrasound

## Abstract

**Purpose:**

This prospective monocentric phase II study (FIDUCOR-study, NCT02526134) aimed to assess the impact of fiducial markers (FMs) implantation on conformal chemo-radiation therapy (CRT) planning in oesophageal carcinoma (EC) patients.

**Methods/materials:**

Fifteen EC patients were enrolled in the study. Each patient underwent two simulation CT-scans before (CT1) and after (CT2) FMs implantation, in the same position. FMs (3 mm length gold markers, preloaded in a 22G needle) were implanted after sedation, under endoscopic ultrasound (EUS) and X-Ray guidance, and were placed at the tumor’s extremities, and in the visible lymph nodes. Target delineation and treatment plan were both performed first on CT1 with the assistance of diagnosis CT, gastroscopy and EUS details, and second on CT2 using FMs and CT-data. The value of FMs implantation was assessed by the difference of growth-tumor-volume (GTV) and clinical-target-volume (CTV) between CT1-based and CT2-based delineation. A significant difference was defined as a ≥5 mm-difference on axial(x) or coronal(y) slices, a ≥10mm-difference on sagittal slices, or a ≥20%-difference in GTV. The impact on dose distribution in organs at risk (OAR) (lung, heart, liver) was also studied.

**Results:**

Between 09/2014 and 12/2015, 15 patients could achieve fiducial procedures, without any complication. One FM migration occurred. We observed a significant modification of the GTV-dimension in 100% of the cases (15/15, 95%CI: [78.2;100.0]), mainly due to a difference in sagittal dimension with a mean variation of 11.2 mm and a difference> 10 mm for 8/15 patients (53.3%). One patient had a significant isocenter displacement as high as 20 mm. The oesophagus tumor was not seen on the CT-scan in one patient due to its small size. One patient had a distant lymph node metastasis not visible on CT-scan. We observed no significant impact on OAR distribution.

**Conclusion:**

In our study, FMs-implantation under EUS had a positive impact on accurate volume definition in EC-patients (modification of GTV in 15/15 patients). Close cooperation between gastroenterologist and radiation oncologist has the potential to improve local treatment of oesophageal carcinoma.

## Introduction

The objective of conformal radiotherapy (CRT) is to improve the dose-distribution, tailored to the target volume borders while reducing the dose to healthy tissues. Accurate delineation of the tumor volume and involved mediastinal lymph nodes is crucial. Computed tomography (CT)-scan is currently the conventional imaging-modality used in Intensity-modulated radiotherapy treatment planning. However, CT does not always allow to distinguish the proximal and distal boundaries between malignant esophageal tumor and healthy esophageal tissues, often because of a poor image-resolution or because the tumor volume can be confused with dietary stasis (1), CT is also not well suited to determine mediastinal lymph node involvement.

For target volume-definition enhancement, radiotherapists often take advantage of image-registration techniques, mixing various image-exams, especially 18F-fluoro-deoxy-2-d-glucose positron emission tomography (FDG-PET-CT), affording good help in RT-treatment planning and eventually in esophageal carcinoma management (2). Another technique to improve target-definition refers to endoscopic ultrasound (EUS)-assisted fiducial markers (FMs) implantation (3–5).This technique was shown feasible without major adverse-events in many different primary cancers, (i.e., lung, pancreas, prostate cancers (3–5), as well as oesophagus carcinomas. The impact on the targeting of radiotherapy has been described for the prostatic cancer irradiation (5, 6). The placement of FMs was also described for digestive tumors in many lesions such as lymph nodes, esophagus, stomach, pancreas, and biliary tract (3, 7–12).

However, the utility of this technique for target volume-definition in radiation therapy (RT) for lesions of the digestive tracts needs investigations. The present study aimed at reporting our findings on the impact of FMs implantation on target volumes-definition in EC-patients considered for definitive or preoperative RT, with or without concurrent chemotherapy. FIDUCOR study, described here, is a non-randomized, monocentric phase II trial, studying the difference in radiotherapy treatment planning before and after EUS-guided fiducial placement.

## Patients and methods

### Patient and tumor characteristics

This single-institution clinical study was conducted between September 2014 and December 2015 in Institut Paoli-Calmettes, Marseille Comprehensive Cancer Center (France). Inclusion criteria were as follows: patients more than 18 years old, referred for radiotherapy +/- concurrent chemotherapy for histologically proven esophageal carcinoma (epidermoid or adenocarcinoma). Previously-treated patients for an esophageal tumor were excluded. At referral, the disease was considered to be limited to the esophagus and regional lymph nodes (except in the case of celiac nodal involvement for a distal esophageal tumor and supraclavicular lymph node involvement for an upper esophageal tumor). Patients whom the tumor could not be crossed by the endoscope, and/or had coagulation disorders and/or portal hypertension were excluded from the study. This protocol was approved by an independent ethics committee, and all the patients had to give written informed consent according to all required guidelines (EudraCT number: 2013-A00916-39; NCT02526134).

The pretreatment evaluation included physical examination, complete blood count, biochemistry surveys of liver and kidney function, esophago-gastroscopy with tumor biopsy, chest and abdominal CT-scan, PET-CT-scan, an ear-nose-throat exam for epidermoid tumors, and EUS of the esophagus.

### EUS-guided fiducial placement

In the case of non-distant lymph nodes highlighted by EUS, EUS-guided fiducial placement was performed. The gold linear fiducial markers (EchoTip Ultra-Fiducial-Needle (ETUF), COOK-Medical Laboratories) measuring 5 mm in length 0.64 mm diameter, were positioned under EUS and X-ray control, in an intubated and supine position patient, using an endoscopic ultrasonography, under general anesthesia. EUS was performed with a slim ultrasound endoscope EG-3270 from Pentax medical. These markers were positioned by the gastroenterologist at the superior and the inferior extremities of the tumor, as well as inside the highest and lowest suspect lymph node if a regional lymph node was present. Fiducial markers were placed submucossally avoiding crossing through the tumor because of the theoretical risk of seeding metastasis. Placing the tumors markers intratumorrally was considered with a risk of migration during the treatment in case of tumoral response.

In the case of distant lymph nodes, a biopsy was performed during the EUS-tumor evaluation. EUS-guided fiducial implantation was performed in a second session, as described above, and a fiducial was also placed in the distant lymph node in case of proven metastasis on pathological reading.

### CT images

Once enrolled in the study, all patients underwent two simulation CT-scans (GE optima 580 RT CT-scan) in the same position, in quiet breathing, before (CT1) and after (CT2) FMs implantation. Patients were placed in the supine position using a neck–rest, arms above the head, and lying on a Symmed arm-rest. The alignment was first performed clinically, using the usual anatomic landmarks and three perpendicular laser beams installed in the room. All patients received intravenous injection according to a standard protocol: 110 mL of non-ionic iodine contrast agent 110s before image acquisition. After checking the patient’s position on anterior-posterior (A/P) and lateral scoot films, we performed a spiral CT-scan using a 1.5 pitch 2.5mm slice thickness, and interslice spacing of 2.5 mm to encompass the entire thorax and upper abdomen. Four ink-points, corresponding to isocenter and alignment, were tattooed on the patient’s chest, at the first simulation-CT time, for positioning purpose.

After FMs implantation, patients underwent the second simulation-CT scan, in the same position using the tattooed marks, and the same window-level settings.

### Volumes definition

Volumes definition were determined according to international delineation guidelines (Wu et al., Créhange G et al.) (13, 14). The Gross Tumor Volume (GTV) was split in GTV-T, corresponding to the esophageal GTV, and GTV-N, corresponding to mediastinal metastatic nodes (defined as nodes with increased FDG uptake or with a short axis of 10 mm in diameter on CT), (GTV= GTV-T + GTV-N).

**Figure 1:**
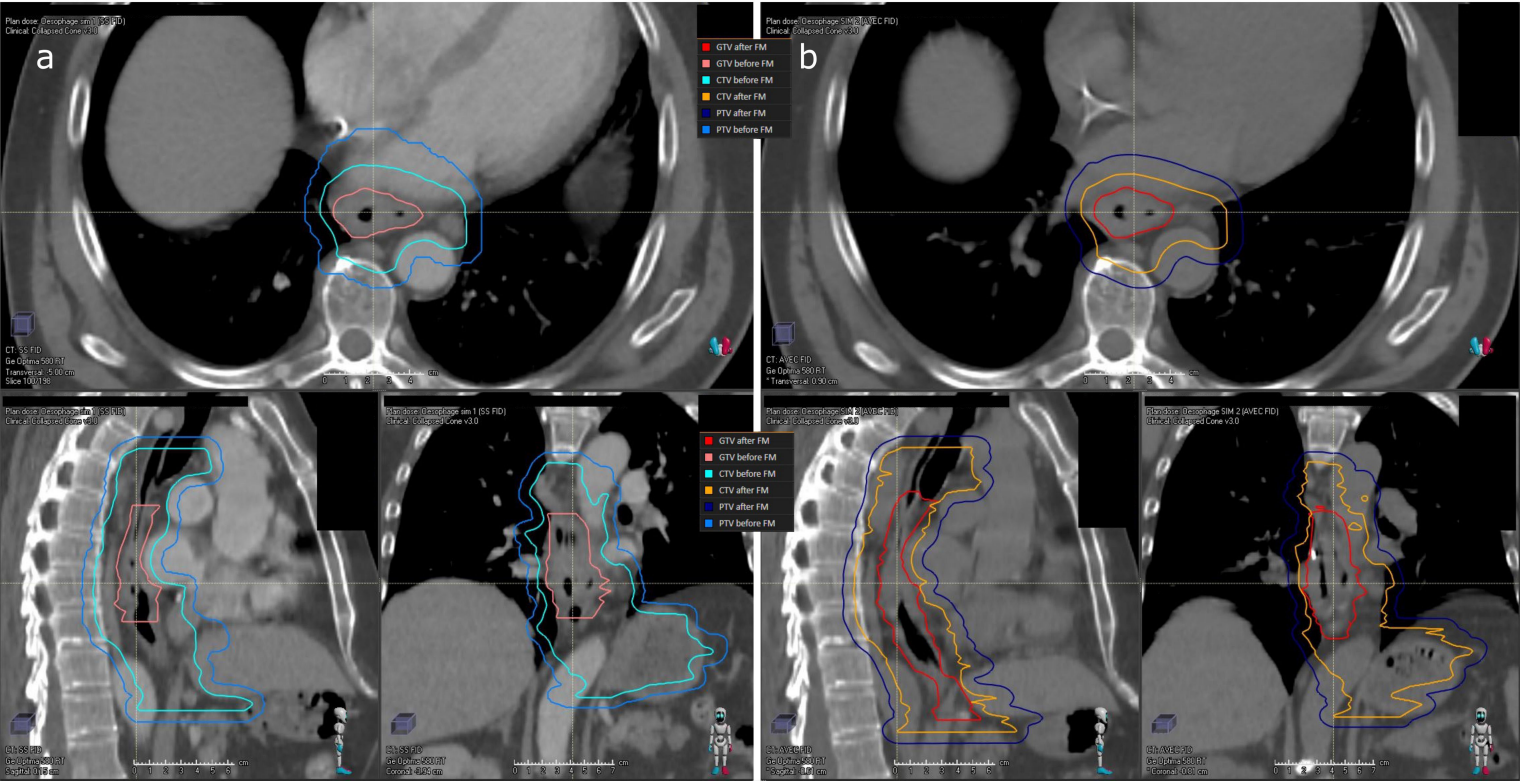
Delineation of target volumes before and after fiducial markers placement for patient n°10. **(A)** on CT1: GTV (pink line) CTV1 (light blue line) PTV1 (medium blue line); **(B)** on CT2: GTV (red line) CTV1 (orange line) PTV1 (dark blue line).

**Figure 2:**
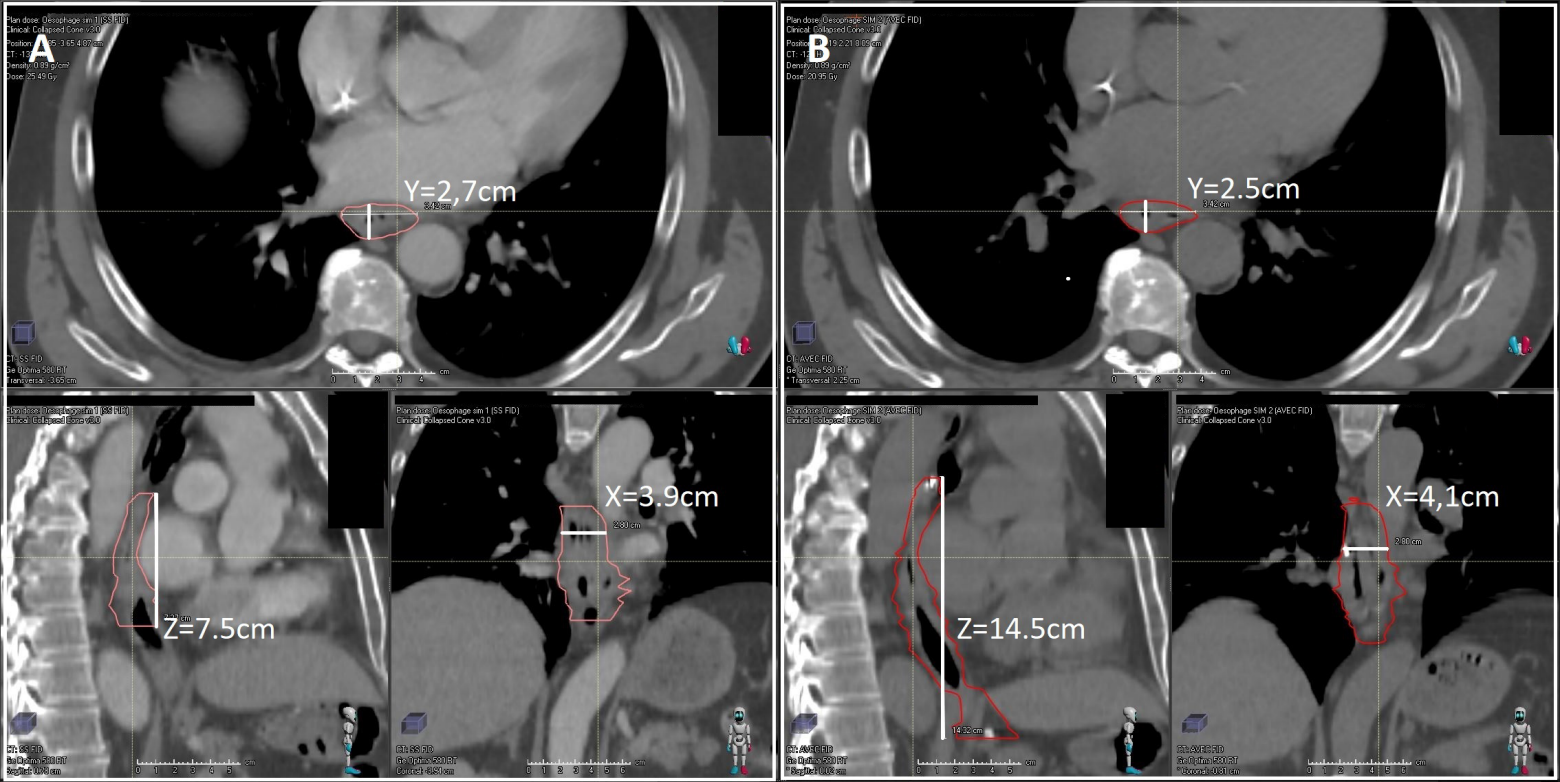
Example of measurement of GTV, CTV, and PTV in X (right - left dimension) or Y (anterior - posterior dimension), or Z (cranio-caudal dimension). for patient n°10 on CT1 **(A)** and B on CT2 **(B)**.

Two Clinical Target Volumes (CTV) were defined: (1) the CTV1, encompassing the GTV and a volumetric margin of 4-5 cm in the cranial-caudal axis, and 1-1.5cm in the radial plan limited to anatomical frontiers (lung, heart, sternum, vertebra, big vessels), to account for microscopic tumor extension (**Figure 1**); (2) the CTV2, encompassing the GTV and a volumetric margin of 2cm in the cranial-caudal axis, and 1-1.5cm in the radial plan limited to the same anatomical frontiers. The margin of 4-5 cm applied in cranio-caudal axis for CTV definition is justified by the risk of longitudinal contiguous sub-epithelial spreading and intramural metastasis of oesophageal cancers. This margin is recommended by both published French and International expert consensus contouring guidelines cited above.

Planning Target volumes (PTV1 and PTV2) were obtained by expanding CTV1 and CTV2, respectively, by a 0.7-1cm isotropic margin to account for mean tumor motion. As recommended by delineation guidelines (Wu et al., Créhange G et al.), pathological locoregional lymph nodes metastases (biopsy-proven) were included in the target volumes for treatment planning (13, 14).

All these volumes, as well as Organs At Risk (OARs) (lungs, esophagus, heart, and spinal cord), were delineated on both CT1 and CT2 as described above by the same radiation oncologist to avoid inter-observer variability. Target volumes delineation on CT1 was guided by the diagnosis-CT scan, the gastroscopy- and EUS-details, while target volumes delineation on CT2 was FMs and CT-data-guided.

### Measures

We compared GTV, CTV, and PTV from CT1 and CT2 by measuring these volumes using the three dimensions: X (right - left dimension), Y (anterior - posterior dimension), and Z (cranial-caudal dimension) (**Figure 2**). The largest size of each was the one retained, and the total volumes were also recorded.

Treatment planning was performed according to ICRU Report 83 (15). The dose was prescribed to the ICRU reference point with lung inhomogeneity corrections. The plans were optimized to maximize the PTV dose while lowering the dose to healthy tissue. The PTV was intended to receive a least 95% and at most 107% of the prescribed dose to 98% and 2% of the PTV, respectively. PTV1 was administered a total dose of 39.6–40 Gy, in 1.8-2 Gy-daily fraction, five fractions a week. PTV2 received an additional boost dose with the same fractionation schedule to ensure a mean total dose of 45-60 Gy.

We used the Pinnacle 9.2 treatment planning system (TPS) for all volume delineations and isocenter positioning, then transferred the data to the Ray-station TPS for the plan dose calculation. The calculation of dose distribution was systematically performed for each treatment plan. The cumulative dose/volume histogram was calculated separately for the GTV and OAR, respecting the following constraints (whatever the reference simulation-CT): the maximum radiation dose (Dmax) to the spinal cord did not exceed 45 Gy (16), the maximal percentage of lungs receiving 20 Gy (V20) was not more than 30% (16). We also compared the following variables: V5 (for the lung), V20 and V30 (for the lung, the liver, and the heart), and V40 (for the heart between the CT1- and CT2-based treatments plans.

Intensity modulated radiation therapy (IMRT) treatments were delivered by an Elekta Synergy linear accelerator using 6-MV photon beams. Half of the patients were treated using six coplanar beam angulations with a maximum of 60 segments per treatment (step and shoot IMRT). For the others, the dose was delivered by two full volumetric modulated arcs (VMAT).

### Outcomes and statistical analysis

The main objective was the rate of significant change, estimated by the number of observed cases divided by the number of evaluated patients. A 95% confidence interval was calculated on the estimated proportion. As regards the results of a precedent publication focused on the impact of the TEP-CT on the radiation volumes in esophagus cancer (2), the difference between the two CT-scans was considered as significant if one or several of the following criteria was observed: i) difference ≥ 5 mm in X (right - left dimension) or Y (anterior - posterior dimension); ii) difference ≥ 1 cm in Z (cranial-caudal dimension); iii) difference of > 20% of the GTV. Secondary objectives were: the direction of GTV variation (increased or decreased size) between CT1-based and CT2-based delineation, the magnitude of isocenter displacement, the difference in dose received by OARs, and FMs implantation adverse-events. Efficacy outcomes of radio-chemotherapy were not assessed in this study.

Data were presented by descriptive statistics. Qualitative variables were summarized by frequencies and percentages. Quantitative data were summarized using position (mean, median) and variability (standard deviation, range) statistics. The statistical analysis was carried out using SAS version 9.3 (SAS Institute, Cary, NC, USA).

## Results

### Fiducial markers implantation procedure

Fifteen EC-patients were enrolled in the study and could achieve fiducial implantation procedures. At least one FM was successfully implanted both at the upper and lower limits of the tumor (range: 1-2, in the two locations) in all patients, while 0 to 4 FM (median:1) were implanted in suspicious lymph nodes. No complication occurred during and after the procedure. For one patient, we observed the migration of one FM, without medical complication.

### Impact on target volume delineation

The mean GTV volume was 57.3 cm^3^ (median: 50.7; range: [5.0-152.4]) without FMs and 77.0 cm^3^ (median: 60.2; range: [8.7-225.7]) with FMs. In one patient, the GTV (GTV-T + GTV-N) significantly decreased with the use of FMs, with a mean decrease of 26.4 cm^3^, whereas for ten patients, the use of FMs for delineation significantly increased the GTV, with a mean increase of 71.4 cm^3^ (median: 57.4; range: [24.7-175.1]). In one patient, the GTV increased because the FMs placement allowed the discovery of an occult positive node in the mediastinum. In another patient, with a T1N0 lesion, the tumor, too small to be visible on CT1, was detected by EUS, and FMs implantation allowed its correct delineation. A 2cm- displacement of the isocenter in the cranial-caudal dimension was observed in one patient. Overall, the GTV was significantly modified by the use of FMs in the cranial-caudal dimension, with a mean difference of 28.9 mm (standard deviation: 21.9) between the two CT-scan. The mean length of the GTV (including macroscopic nodes) without FMs use was 72.9 mm (median: 70.0; range: [27.5–166.2]) vs. 94.5 mm with FMs use (median: 85.0; range: [42.5–142.5]).

The mean length of the esophageal tumor (GTV-T) without FMs use was 61.0 mm (median: 57.5; range: [27.5–112.5]) *vs.* 69.2 mm with FMs use (median: 65.0; range: [30.0–140.0]). A reduction of the esophageal tumor length was observed in 3 patients (mean: 31.4 mm, median: 17.0, range:[12.5-64.7]). An esophageal tumor-length increase was observed in 6 patients (mean increase: 30.7 mm, median: 27.0, range: 16.5-63.2). Details of GTV modifications for each patient are given in **Table 1**.

**Table 1:** Gross tumor volume (GTV) modifications before and after fiducial markers (FM) in the FIDUCOR cohort (n=15).

	GTV dimensions before/after FM: increase (+) or decrease (-) in mm			GTV volume before/after FM (cc): increase (+) or decrease (-), %	Significant yes/no (significant axis)
Patient	Left-right (x)	Anterior-posterior (y) :	Superior-inferior (z)	Volume	
1	41/41: 0	33/34: +1	**104/87: -17**	72/60: -17%	Yes (z)
2	**30/24: -6**	23/19: -4	**73/83: +10**	20/21: +5%	Yes (x,z)
3	36/40.5: +4.5	22/24: +2	139/130: -9	**16/44: +175%**	Yes (volume)
4	43/46: +3	41/32: -9	**45/88: +43**	38/43: +16%	Yes (z)
5	44/40: -4	60/60: 0	**38/14: -24**	**49/66: +35%**	Yes (z,volume)
6	19/19: 0	**14/21: +7**	**25/52: +27**	**50/87: +74%**	Yes (y, z,volume)
7	**69/62: -7**	**60/65: +5**	98/96: -2	141/140: -0.7%	Yes (x,y)
8	**53/58: +5**	**40/61: +21**	**56/44: -12**	**51/119: +133%**	Yes (x,y, z,volume)
9	**31/25: -6**	**26/36: +10**	**36/59: +23**	**23/34: +48%**	Yes (x,y, z,volume)
10	39/41: +2	27/25: -2	**75/145: +70**	**36/60: +67%**	Yes (z,volume)
11	32/31: -1	28/24: -4	63/60: -3	**51/38: -25%**	Yes (volume)
12	52/52: 0	37/37: 0	**57/74: +16**	**86/109: +27%**	Yes (z,volume)
13	38/42: +4	43/39: -4	**51/115: +64**	**67/123: +81%**	Yes (z,volume)
14	40/40: 0	25/24: -1	**106/136: +30**	**52/65: +25%**	Yes (z,volume)
15	**49/64: +15**	45/48: +3	**117/144: +27**	**152/225: +48%**	Yes (x, z,volume)

Bold values are statistically significant values.

### Impact on the total dose delivered to the organ at risk

Dosimetric comparison of Organs at risk (OARs) dose distribution before and after fiducial markers (FMs) placement is presented in **Table 2**. The mean percentage of lung receiving 5 Gy (V5), 20 Gy (V20) and 30 Gy (V30) was 67.7% (range: [31.2-97.5]), 16.5% (range: [2.0-30.7]) and 5.8% (range: [0.74-11.9]), respectively, before FMs placement versus 66.8% (range: [28.8-91.8]), 18.0% (range: [3.1-34.0]), and 5.9% (range: [0.9-14.1]), respectively, after FMs placement. The mean dose (Dmean) delivered to the lungs was 11.8 Gy (range: [4.2-17.2]) before FMs placement versus 11.7 Gy (range: [4.5-16.4]) after FMs placement.

**Table 2:** Dosimetric comparison of Organs at risk (OARs) dose distribution before and after fiducial markers (FMs) placement.

OAR	Dosimetric parameter	Mean values before FMs placement	Mean values after FMs placement
LUNGS	V5 (% [range])	67.7 [31.2-97.5]	66.8 [28.8-91.8]
	V20 (% [range])	5.8 [0.74-11.9]	18.0 [3.1-34.0]
	V30 (% [range])	16.5 [2.0-30.7]	5.9 [0.9-14.1]
	Dmean (Gy [range])	11.8 [4.2-17.2]	11.7 [4.5-16.4]
HEART	V20 (% [range])	41.4 [20.3-85.0]	40.7 [25.8-65.0]
	V30 (% [range])	20.0 [7.6-43.7]	18.6 [11.2-30.2]
	V40 (% [range])	10.3 [3.3-22.5]	8.9 [0.7-19.6]
	Dmean (Gy [range])	20.3 [12.3-31.1]	19.7 (15.4-26.3]
	Dmax (Gy [range])	49.4 [45.6-61.6]	48.7 [39.8-59.7]
LIVER	V20 (% [range])	24.8 [0.0-44.9]	28.9 [0.0-56.1]
	V30 (% [range])	15.5 [0.0-77.7]	11.6 [0.0-22.8]
	Dmean (Gy [range])	13.3 [0.2-20.4]	13.7 (0.7-21.7]

Mean values and range are reported.

The mean percentage of heart receiving 20 Gy (V20), 30 Gy (V30) and 40 Gy (V40) was 41.4% (range: [20.3-85.0]), 20.0% (range: [7.6-43.7]) and 10.3% (range: [3.3-22.5]), respectively, before FMs placement versus 40.7% (range: [25.8-65.0]), 18.6% (range: [11.2-30.2]), and 8.9% (range: [0.7-19.6]), respectively, after FMs placement. The mean of the mean and the maximum dose delivered to the heart was respectively 20.3 Gy (range: [12.3-31.1]) and 49.4 Gy (range: [45.6-61.6]) before FMs placement versus respectively 19.7 Gy (range: (15.4-26.3]) and 48.7 Gy (range: [39.8-59.7]) after FMs placement.

The mean percentages of liver receiving 20 Gy (V20) and 30 Gy (V30) were 24.8% (range: [0.0-44.9]), and 15.5% (range: [0.0-77.7]), respectively, before FMs placement versus 28.9% (range: [0.0-56.1]), and 11.6% (range: [0.0-22.8]), respectively, after FMs placement. The mean dose delivered to the liver was 13.3 Gy (range: [0.2-20.4]) before FMs placement versus 13.7 Gy (range: (0.7-21.7]) after FMs placement.

## Discussion

An optimal dose delivery, avoiding geographic misses, and reducing the volume of healthy tissue irradiated, would ensure maximal tumor local control, without compromising the quality of life. Current standard practice in EC volume definition, in the setting of curative intent external beam CRT, relies on the combination of CT and PET-CT datasets (16–18). FDG-PET success in identifying most primary tumors, with a 30-93% sensitivity (19, 20) and a 79-100% specificity (21) for the detection of metastatic lymph nodes. In a prospective study comparing PET-scan, CT-scan and ultrasonography in the diagnosis of esophageal and esophagogastric junction cancers, was shown of limited value due to a weak accuracy in para-tumoral and distant lymph nodes staging of. Although PET-scan was proven superior to CT-scan in metastases detection (22), low evidence supports its use in tumor delineation, mainly explained by the inflammation surrounding the tumor, leading to false-positive uptakes (23).

On the other hand, the use of FMs to enhance RT-volume definition is currently validated and routinely used in prostate cancer irradiation (4–6). Fiducials implant was also still described in many digestive tumors such as lymph nodes, esophagus, stomach, pancreas, and biliary tract, but never used to guide radiotherapy (3, 7–12, 24). Endoscopic placement of fiducial markers for radiation therapy guidance is a relatively newer application of EUS in pancreatic and thoracic tumors. One report described the successful use of fiducials placed under linear EUS guidance only in patients with abdominal and mediastinal tumors, but this report did not address EC-patients (3).

Our study is a prospective monocentric study, conducted in a short period, avoiding the potential bias of inhomogeneity between different teams for the placement of FM. Furthermore, target volume delineation was done by the same radiotherapist, both before and after FM placement, avoiding inter-observer delineation variability. Moreover, the radiotherapist strictly respected the rules to measure the largest dimension for each tumor, to reduce the risk of intra-operator variability.

In this study, and according to our main objective criteria (composite parameter), we observed that FM implantation significantly modified the GTV in 15/15 patients (100%, CI 95%: [78.2-100.0]), mainly due to an increase in this volume (10/15, 66.7%). A GTV modification was mainly observed in the cranial-caudal dimension, being statistically significant in 8/15 patients (53.3%). FMs implantation also led to the discovery of occult lymphadenopathy in one patient, EUS being already well-described in the literature as an efficient tool for occult-distant lesions detection (25, 26). More interesting was the case of one patient medically unfit for surgery and harboring a too small lesion to be visible on both the CT and the PET-CT-scans (only visualized by endoscopy): in whom only FMs placement made target volume definition possible.

Whereas FMs-driven volume modification appears of lower magnitude in PTV and has little impact on OARs dose distribution, the increasing use of hypofractionated radiotherapy schedules, with higher dose-fractionation and lower margins around the GTV, makes FMs implantation an attractive method for the accurate definition of RT-target volumes.

However, our study presents some limitations. First, the small sample size requires confirmation of the findings through a prospective study, currently ongoing in France (FIDECHO). Second, this technique is feasible only in the case of tumors that can be easily crossed by endoscopy. We used in this study a slim ultrasound endoscope (EG-3270UK from Pentax medical^®^), thinner than usual echo-endoscopes, which allowed to cross all the esophagus tumors. Noteworthy, fiducials also had the advantage of their radio-opacity, lowering patient setup errors during the treatment course and also reducing healthy tissue (pulmonary, cardiac and esophageal) irradiation. However, the aim of our study was not to demonstrate such an interest, and this potential advantage might be investigated in specific study. Finally, in this small size cohort, we chose not to report efficacy outcomes of CRT (already available in the literature) to focus on the impact of FMs on definition of RT-target volumes.

To our knowledge, this is the first study to demonstrate the potential interest of this technique in the definition of RT-target volume in digestive tract lesions, especially in esophagus radiotherapy. These data will be expanded by the ongoing larger scale prospective French multicenter FIDECHO study.

## Conclusion

This study suggested a positive-impact of FM the implantation for the definition of RT-target volumes in esophagus radiotherapy. A larger scale, prospective multicenter study is currently underway to validate our preliminary data.

## Data availability statement

The raw data supporting the conclusions of this article will be made available by the authors, without undue reservation.

## Ethics statement

The studies involving human participants were reviewed and approved by CPP Sud Méditerranéen, réf 1373. The patients/participants provided their written informed consent to participate in this study. Written informed consent was obtained from the individual(s) for the publication of any potentially identifiable images or data included in this article.

## Author contributions

Conception of the study: LM-Z, FC. Endoscopic procedures: FC, JR. Collection of the data: LM-Z, FC, LLA. Data analysis: LM-Z, FC, LLA. Writing – original draft: LM-Z, PR, MT. Writing – review & editing: all authors. All authors contributed to the article and approved the submitted version.

## Funding

This work is supported by Institut Paoli-Calmettes.

## Acknowledgments

We would like to thank the patients who accepted to take part to this research.

## Conflict of interest

The authors declare that the research was conducted in the absence of any commercial or financial relationships that could be construed as a potential conflict of interest.

## Publisher’s note

All claims expressed in this article are solely those of the authors and do not necessarily represent those of their affiliated organizations, or those of the publisher, the editors and the reviewers. Any product that may be evaluated in this article, or claim that may be made by its manufacturer, is not guaranteed or endorsed by the publisher.

## Abbreviations

CRT, Conformal Chemo-Radiation; CTV, Clinical Target Volumes; EUS, Endoscopic Ultrasound; GTV, Gross Tumor Volume; FM, Fiducial Markers; OAR, Organs At Risk.
